# Evaluation of Sonicate Fluid Culture Cutoff Points for Periprosthetic Joint Infection Diagnosis

**DOI:** 10.1093/ofid/ofae159

**Published:** 2024-03-20

**Authors:** Judith Alvarez Otero, Melissa J Karau, Kerryl E Greenwood-Quaintance, Matthew P Abdel, Jay Mandrekar, Robin Patel

**Affiliations:** Division of Clinical Microbiology, Department of Laboratory Medicine and Pathology, Mayo Clinic, Rochester, Minnesota, USA; Division of Clinical Microbiology, Department of Laboratory Medicine and Pathology, Mayo Clinic, Rochester, Minnesota, USA; Division of Clinical Microbiology, Department of Laboratory Medicine and Pathology, Mayo Clinic, Rochester, Minnesota, USA; Department of Orthopedic Surgery, Mayo Clinic, Rochester, Minnesota, USA; Department of Quantitative Health Sciences, Mayo Clinic, Rochester, Minnesota, USA; Division of Clinical Microbiology, Department of Laboratory Medicine and Pathology, Mayo Clinic, Rochester, Minnesota, USA; Division of Public Health, Infectious Diseases, and Occupational Medicine, Department of Medicine, Mayo Clinic, Rochester, Minnesota, USA

**Keywords:** cutoff points, diagnosis, periprosthetic joint infection, sonicate fluid cultures, sonication

## Abstract

**Introduction:**

Implant sonication is useful for recovery of periprosthetic joint infection (PJI) pathogens in culture, but exact cutoff points for definition of clinically significant sonicate fluid culture results vary from study to study. The aim of this study was to define ideal sonicate fluid culture cutoff points for PJI diagnosis.

**Methods:**

Sonicate fluid cultures from hip and knee prosthesis components removed between February 2007 and December 2020 were studied. Prosthesis components were placed in solid containers in the operating room; in the clinical microbiology laboratory, 400 mL Ringer's solution was added, and containers subjected to vortexing, sonication and then vortexing, followed by centrifugation. Concentrated sonicate fluid was plated on aerobic and anaerobic solid media, and culture results reported semiquantitatively, as no growth, <20, 20–50, 51–100, or >100 CFU/10 mL sonicate fluid. Sonicate cultures from cement spacers and cultures yielding more than 1 microorganism were excluded. Sensitivity and specificity of each cutoff point was evaluated.

**Results:**

A total of 1448 sonicate fluid cultures were evaluated, 68% from knees and 32% from hips. PJI was present in 644 (44%) cases. Sensitivity of sonicate culture was 75.0% at <20 CFU/10 mL, 55.3% at ≥20 CFU/10 mL, 46.9% at >51 CFU/10 mL, and 39.8% at >100 CFU/10 mL. Specificity was 78.2%, 99.8%, 100%, and 100%, at the 4 cutoff points, respectively.

**Conclusions:**

A cutoff point for sonicate fluid culture positivity of ≥20 CFU/10 mL is suitable for PJI diagnosis.

The yearly number of total hip and knee arthroplasty surgeries done in the United States has increased over time and is projected to continue to do so [1–3], with consequent increases in hip and knee periprosthetic joint infection (PJI). PJI is a devastating disease that demands accurate diagnosis to define appropriate therapy. Diagnosis may be a challenge and involves differentiating PJI from noninfectious joint failure (NIAF), and, once a diagnosis is established, defining the microbiology to tailor antimicrobial therapy [4].

Microorganisms involved in PJI are typically attached to the prosthesis surface, where they form biofilms, a process fundamental to the pathogenesis of PJI. Sonication of resected arthroplasty components allows sampling of bacteria on their surfaces using culture [5–7], improving PJI diagnosis compared with periprosthetic tissue cultures [8]. When analyzing fluid derived from implant sonication (sonicate fluid), however, it is important to use appropriate cutoff values to define PJI because small numbers of microorganisms recovered from sonicate fluid may represent contamination. Varying cutoff points have been applied in different studies. The European Bone and Joint Infection Society definition of PJI considers PJI “confirmed” at a cutoff point of 50 CFU/mL (200 CFU/mL if centrifugation is used), “likely” at >1 CFU/mL, and “unlikely” with no growth, but recommends caution with interpretation of values <50 CFU/mL, with consideration of such findings together with other evidence [9]. Other PJI definitions do not address the sonicate fluid cutoff points for PJI [10, 11].

The purpose of this study was to evaluate sonicate fluid culture cutoff points for PJI diagnosis using a large patient cohort.

## METHODS

### Study Population

Sonicate fluid cultures from hip and knee prosthesis components removed between February 2007 and December 2020 for NIAF or presumed infection at Mayo Clinic, in Rochester, Minnesota, were included. Sonicate fluid cultures from cement spacers and hardware other than prosthetic joint components were excluded to focus on infected hip and knee arthroplasty components. Cultures yielding more than 1 microorganism were excluded because it would have been challenging to assess multiple organisms found in different amounts.

### Diagnosis of Prosthetic Joint Infection

A diagnosis of PJI was made if at least 1 of the following was present: A sinus tract communicating with the prosthesis; visibly purulent synovial fluid or visible purulence surrounding the prosthesis; acute inflammation on histopathological examination of permanent tissue sections as determined by a pathologist; or 2 or more intraoperative tissue cultures or a combination of preoperative aspiration culture and an intraoperative culture yielding the same organism [10]. NIAF was defined as failure of a prosthesis absent these criteria.

### Conventional Microbiologic Methods

Intraoperative tissue samples with the most obvious inflammatory changes were collected for histopathological and microbiologic studies. Tissue specimens were homogenized using a Seward Stomacher 80 Biomaster (Seward Inc., Port St. Lucie, FL) in 5 mL of brain–heart infusion broth for 1 minute. Between 2007 and 2015, 0.1 mL of this solution was inoculated onto aerobic and anaerobic blood agar plates and into 1 mL anaerobically prereduced thioglycolate broth (BD Diagnostic Systems, Cockeysville, MD). Aerobic plates were incubated for 5 days and anaerobic plates and broths for 14 days. From 2016 onward, 1 mL of the homogenized solution was inoculated into BACTEC Plus Aerobic/F and BACTEC Lytic/10 Anaerobic/F bottles (BD Diagnostic Systems) and the bottles incubated in a BACTEC FX9240 instrument (BD Diagnostic Systems) for 14 days or until positive [12]. Tissue cultures were considered positive if at least 2 grew the same organism, or if 1 was positive for a virulent microorganism such as *Staphylococcus aureus*. Between 2004 and 2019, synovial fluid was inoculated into a BACTEC Peds Plus/F bottle and incubated on a BACTEC FX 9240 instrument for 5 days if more than 1 mL was received or onto aerobic blood agar, chocolate agar, and CDC anaerobic 5% blood agar, and into thioglycolate broth if less than 1 mL was received, with incubation as per tissue cultures. From 2019 onward, synovial fluid was inoculated into BACTEC Aerobic/F and BACTEC Lytic/10 Anaerobic/F bottles and incubated on a BACTEC FX 9240 instrument for 14 days if more than 2 mL was received or onto aerobic blood agar, chocolate agar, and CDC anaerobic 5% blood agar, and into thioglycolate broth if less than 2 mL was received, with incubation as per tissue cultures.

### Sonication of Removed Prostheses

Prosthesis components were placed in solid containers in the operating room and processed in the microbiology laboratory. In the clinical laboratory, 400 mL Ringer's solution was added, and containers subjected to vortexing, sonication (5 minutes) and then vortexing, followed by centrifugation for 100-fold concentration. Concentrated sonicate fluid was plated to aerobic and anaerobic solid media, and culture results reported semiquantitatively, as no growth, organism present <20, 20–50, 51–100, or >100 CFU/10 mL.

### Isolate Identification

Bacterial isolates were identified using standard laboratory techniques.

### Statistical Analysis

Descriptive summaries are provided as means (standard deviations) or frequencies (percentages). Comparisons between the PJI and NIAF and variables of interest were performed using chi-square or Wilcoxon rank-sum tests, as appropriate. Sensitivity and specificity were estimated for cutoff points of <20, 20–50, 51–100, and >100 CFU/10 mL, with the best cutoff point identified based on the value that maximized the sum of sensitivity and specificity. Further comparisons between variables of interest were performed between the 2 groups based on the cutpoint identified (eg, <20 CFU/10 mL vs ≥20 CFU/10 mL). All tests were 2 sided; *P* values less than .05 were considered statistically significant. Analysis was performed using SAS software version 9.4 (SAS inc, Cary, NC).

## RESULTS

A total of 1746 sonicate fluid cultures were studied; 22 were excluded because they were from hardware-related nonhip or knee arthroplasty components, 171 because they were from cement spacers, and 105 because of more than 1 microorganism being recovered from sonicate fluid. Of 1448 sonicate fluid cultures analyzed, 645 (44%) were from cases classified as PJI and 803 (56%) from cases classified as NIAF. Fifty-one cases were diagnosed as having PJI based on conventional cultures alone (ie, no intraoperative purulence, sinus tract, or any other criteria); of the 51, 47 sonicate fluids yielded positive concordant results, 2 were negative, and 2 yielded discordant positive results.

Demographic and clinical characteristics and laboratory data are shown in Table 1. Clinical presentations before surgery were different between the groups. In the NIAF group, pain (95%) was the most notable clinical finding. In the PJI group, pain (90%) and local findings of infection (58%) were frequently encountered. C-reactive protein and erythrocyte sedimentation rate were higher in PJI compared with NIAF cases (33 vs 3.4 mg/dL, *P* < .0001; and 39 vs 9 mm/h, *P* < .0001, respectively).

**Table 1.** Baseline Cohort Characteristics

Characteristic	Periprosthetic Joint Infection	Noninfectious Arthroplasty Failure	*P*
Age (y)	66	65	.42
Sex (%)			.018
Female	47	53	
Male	53	47	
Body mass index (median)	31	32	.91
Site of arthroplasty (%)			<.0001
Knee	61	71	
Hip	39	29	
Symptoms/signs (%)			
Pain	90	95	.38
Local findings of infection	58	5	<.0001
Presence of a sinus tract	15	0	<.0001
Visible purulence implant site at surgery (%)	63	0	<.0001
Preoperative laboratory findings			
Erythrocyte sedimentation rate (mm/h)	39	9	<.0001
Serum C-reactive protein (mg/dL)	33	3.4	<.0001
Synovial fluid leukocyte count (/μL)	20 476	762	<.0001
Synovial fluid neutrophil percentage (%)	89	15	<.0001

The sensitivity of sonicate culture at a cutoff point of <20 CFU/10 mL was 75.0%, at a cutoff point of ≥20 CFU/10 mL was 55.3%, at a cutoff point of >50 CFU/10 mL was 46.9%, and at cutoff point of >100 CFU/10 mL was 39.8%. The specificities at the 4 cutoff points were 78.2%, 99.8%, 100% and 100%, respectively (Table 2).

**Table 2.** Sensitivity, Specificity, and Positive and Negative Predictive Values of Cutoff Points Evaluated

Cutoff Points	Sensitivity (%)	Specificity (%)	Positive Predictive Value (%)	Negative Predictive Value (%)
Any positive	75.0	78.2	73.4	79.6
≥20 CFU/10 mL	55.3	99.8	99.4	73.6
≥51 CFU/10 mL	46.9	100.0	100.0	70.1
>100 CFU/10 mL	39.8	100.0	100.0	67.4

The most frequent microorganism isolated in sonicate fluid culture was *Staphylococcus epidermidis,* 126 in PJI and 3 in NIAF cases; followed by *S aureus* in 93 PJI (and none in NIAF) cases; streptococci in 46 PJI and 2 NIAF cases; and enterococci in 36 PJI and 2 NIAF cases. There were 15 isolations of *Staphylococcus lugdunensis* (14 in PJI and 1 in NIAF cases), 13 of *Cutibacterium acnes* (11 in PJI and 2 in NIAF cases), 11 of Enterobacterales (9 in PJI and 2 in NIAF cases), and 11 of *Pseudomonas aeruginosa* (all from PJI cases).

**Figure 1.**
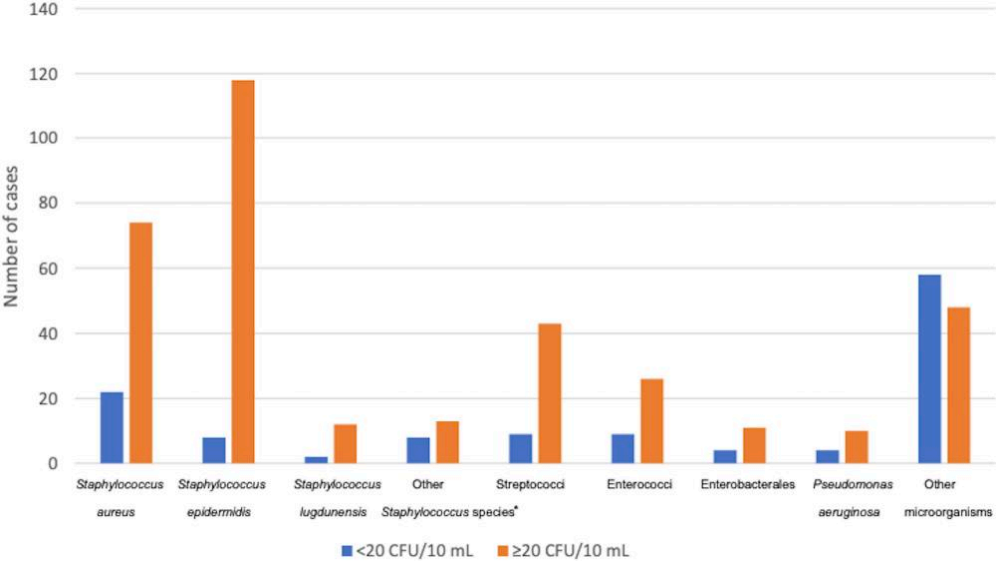
Microbiology by quantity in periprosthetic joint infection cases. *Other *Staphylococcus* species: *Staphylococcus* species other than *Staphylococcus aureus*, *Staphylococcus epidermidis*, and *Staphylococcus lugdunensis*.

**Figure 2.**
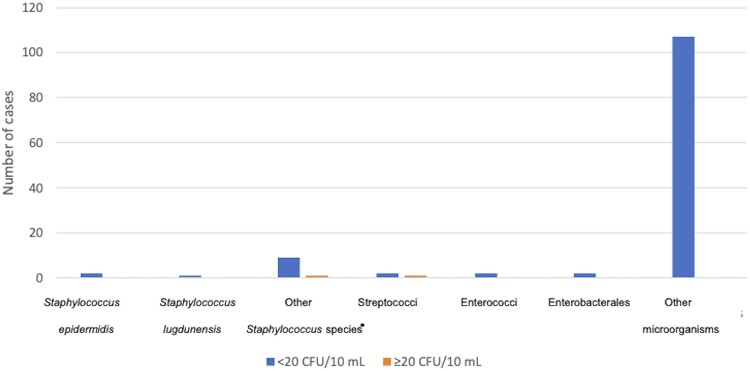
Microbiology by quantity in noninfectious arthroplasty failure cases. *Other *Staphylococcus* species: *Staphylococcus* species other than *Staphylococcus aureus*, *Staphylococcus epidermidis*, and *Staphylococcus lugdunensis*.

*S aureus* was recovered at <20 and ≥20 CFU/10 mL in 22/645 and 74/645 PJI cases (and was not found in any NIAF case). *S epidermidis* was isolated at <20 and ≥20 CFU/10 mL in 8/645 and 118/645 PJI cases and 2/803 and 0/803 NIAF cases. *S lugdunensis* was isolated at <20 and ≥20 CFU/10 mL in 2/645 and 12/645 PJI cases and 1/803 and 0/803 NIAF cases. *Staphylococcus* species other than *S aureus, S epidermidis,* and *S lugdunensis* were isolated at <20 and ≥20 CFU/10 mL in 8/645 and 13/645 PJI cases and 9/803 and 1/803 NIAF cases. Streptococci were isolated at <20 and ≥20 CFU/10 mL in 9/645 and 43/645 PJI cases and 2/803 and 1/803 NIAF cases. Enterococci were isolated at <20 and ≥20 CFU/10 mL in 9/645 and 26/645 PJI cases and 2/803 and 0/803 NIAF cases. Enterobacterales were isolated at <20 and ≥20 CFU/10 mL in 4/645 and 11/645 PJI cases and 2/803 and 0/803 NIAF cases. *P aeruginosa* was isolated at <20 and ≥20 CFU/10 mL in 4/645 and 10/645 PJI cases and no NIAF cases. Other microorganisms were isolated at <20 and ≥20 CFU/10 mL in 58/645 and 48/645 PJI cases and 107/803 and 0/803 NIAF cases (Figures 1 and 2).

## DISCUSSION

Results of this study indicate a sonicate fluid culture cutoff of ≥20 CFU/10 mL to be appropriate for diagnosis of hip and knee PJI, when using a concentration step after sonication. In 2007, our group described a cutoff point for unconcentrated sonicate fluid positivity for hip and knee implants of 5 CFU/plate, with 0.5 mL sonicate fluid plated [5]. In this study, a 100-fold concentration step was used, and 0.1 mL was plated instead of 0.5 mL, justifying redefining of the cutoff point. Plating of 0.5 mL was an amount that required prolonged plate drying, a challenge overcome using a plating volume of 0.1 mL.

Beyond our original study [5], and the results reported here, few studies have specifically evaluated sonicate culture cutoff points for PJI diagnosis. Rothenberg et al. reported that the optimal cutoff point was 5 CFU/agar plate for hip and knee prosthesis sonicate fluids (using 400 mL lactated Ringer's solution, centrifugation, and a final plating volume of 0.1 mL) [13]. In another study involving hip and knee prostheses, the sensitivity of sonicate fluid culture with a cutoff point of 50 CFU/mL was 77%, and the sensitivity of conventional tissue cultures was 56% (*P* value, .012); the specificities of the 2 methods were 98% and 94%, respectively [8]. A different study showing that sonicate cultures were more sensitive than tissue cultures used a cutoff point of 50 CFU/mL [14]. Studies performed to evaluate molecular diagnostics applied to sonicate fluid have considered ≥20 CFU/10 mL as significant results [15, 16]. Ueda et al. evaluated sonication of orthopedic implants involving several joints-types and defined detectable levels of microorganisms using a cutoff of 0.1 CFU/mL; sensitivity and specificity were 71% and 100%, respectively [17]. In a study on prosthetic shoulder sonicate fluids derived using the concentration process described here, most infections (12 of 22 with positive cultures) were associated with sonicate fluid culture growth of ≥100 CFU per plate, with 7 yielding 20 to 99 CFU per plate and 3 yielding less than 20 CFU per plate [18]. In a study on prosthetic shoulder infection performed by Grosso et al., sensitivity and specificity were 56% and 93%, respectively, with a cutoff of >20 CFU/mL, whereas with no cutoff value, sensitivity and specificity were 96% and 64%, respectively [19]. Another study showed that sensitivity and specificity of sonicate fluid culture with a cutoff of 50 CFU/mL were 36% and 98%, respectively; and 75% and 82% with no cutoff point [20].

Holinka et al. defined sonicate fluid positivity as at least 3 CFUs of the same organism on any plate; implant components were separately sonicated in an attempt to assess the distribution of microorganisms on different implant components; cups of hip prostheses and tibial components of knee prostheses had the highest detection rates [21]. Portillo and collaborators reported that with a cutoff of ≥1 CFU/mL, sensitivity of sonication combined with vortexing was 71% and specificity 92%; at a cutoff of ≥50 CFU/mL, sensitivity was 60% and specificity 99% [22]. A metaanalysis of sonicate fluid cultures, which included various implant types, suggested an ideal cutoff point to be ≥5 CFU. However, studies that used varied sonication methods (vortexing in 3 and centrifugation in 5 of 12 studies), and plate incubation periods (eg, anaerobic cultures were incubated for 7 or 14 days) were included [23]. Another metaanalysis that also had significant heterogeneity among the studies [24] showed similar results.

This work has several limitations. It was a retrospective study performed at a single center, using a defined (but specific), standardized method to perform sonicate fluid cultures, consisting of vortexing, sonication and vortexing, followed by centrifugation. Only hip and knee PJIs were evaluated. Polymicrobial infections and cement spacers were excluded. Further studies should evaluate cutoff points for sonication of cement spacers and polymicrobial infections. The sensitivity of sonication culture reported here is lower than reported in our previous study [5–7], likely because of differences in patient populations; the prior study prospectively enrolled subjects to evaluate diagnostic accuracy of sonication culture, whereas this study included clinically ordered sonication cultures, possibly preferentially requested in those with known negative synovial fluid and/or prior tissue culture results.

This study shows that a sonicate fluid cutoff point is necessary so as not to yield nonspecific findings, and that, overall, a cutoff point of ≥20 CFU/10 mL is suitable for hip and knee PJI diagnosis using the described implant sonication approach. Results of this study should be helpful to those interpreting results of sonication culture.
