# Correction to: Evaluation of Sonicate Fluid Culture Cutoff Points for Periprosthetic Joint Infection Diagnosis

**DOI:** 10.1093/ofid/ofae300

**Published:** 2024-05-30

This is a correction to: Judith Alvarez Otero, Melissa J Karau, Kerryl E Greenwood-Quaintance, Matthew P Abdel, Jay Mandrekar, Robin Patel, Evaluation of Sonicate Fluid Culture Cutoff Points for Periprosthetic Joint Infection Diagnosis, *Open Forum Infectious Diseases*, Volume 11, Issue 5, May 2024, https://doi.org/10.1093/ofid/ofae159

The following changes have been made to the originally published paper.

In Table 1 the *p*-value for Sex has been corrected from .08 to .018, and the value for Site of arthroplasty from .08 to <.0001.

In the **Results** section the statement ‘No differences were found in age and sex between the 2 groups.’ has been removed.

